# Two cases of ectopic extramammary Paget’s disease

**DOI:** 10.1016/j.jdcr.2025.02.033

**Published:** 2025-03-14

**Authors:** Jonathan Banta, Carolyn Hardin Robinson, William Schaffenburg

**Affiliations:** a Dermatology Resident, Department of Dermatology, Wilford Hall Ambulatory Surgical Center, Lackland AFB, Texas; b Staff Dermatologist and Mohs Surgeon, Department of Dermatology, Wilford Hall Ambulatory Surgical Center, Lackland AFB, Texas; c Dermatologist and Dermatopathologist, Department of Dermatology, Wilford Hall Ambulatory Surgical Center, Lackland AFB, Texas

**Keywords:** ectopic extramammary Paget's disease, Mohs, dermatopathology, pagetoid

## Introduction

Extramammary Paget’s disease (EMPD) is a rare adenocarcinoma that primarily affects areas of the skin with a high presence of apocrine glands.^1^, ^2^, ^3^, ^4^, ^5^, ^6^, ^7^, ^8^, ^9^ In contrast, ectopic extramammary Paget’s disease (eEMPD) is a rarer form of EMPD that appears on areas of the skin that typically lack the presence of apocrine glands.^1^ In their 2019 review, *Scarbrough et al* reported a total of 45 cases of eEMPD, with only 4 cases on the face and 6 cases on the extremities.^1^ Herein, we present 2 novel cases of eEMPD—one on the posterior arm and another on the face that was initially diagnosed as melanoma in situ. These cases highlight the need for clinical awareness and diagnostic scrutiny to provide appropriate diagnoses and better patient outcomes.

## Case report 1

**Fig 1:**
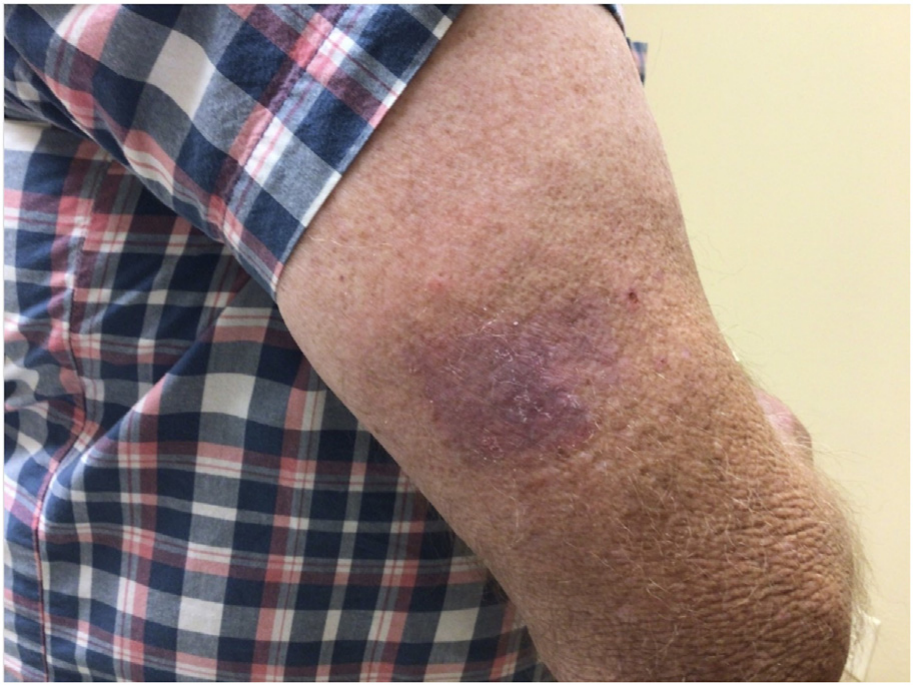
A 4 cm × 3 cm erythematous-violaceous plaque located on right posterior upper arm from Case 1.

**Fig 2:**
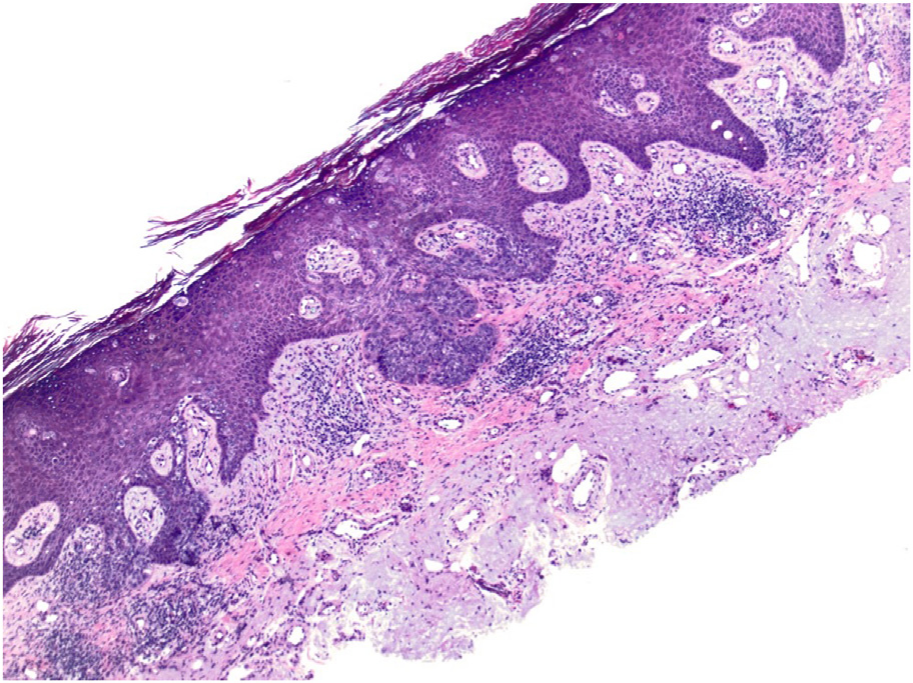
Shave biopsy from Case 1 showing a hyperplastic epidermis populated by large, pale, “pagetoid” cells with atypical nuclei extending into adnexal structures (H&E 20×).

**Fig 3:**
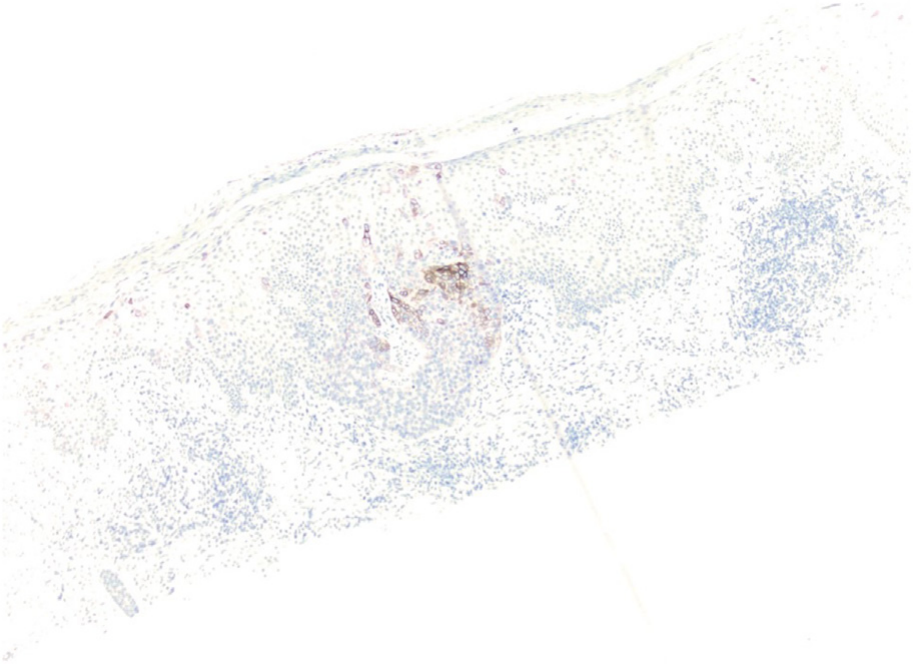
Immunohistochemical staining from Case 1 showing positivity for cytokeratin 7 (CK7).

A 72-year-old male with Fitzpatrick skin type II presented with a persistent 4 cm × 3 cm erythematous-violaceous plaque on his right posterior upper arm, featuring pruritus, redness, and serous drainage of a 4-month duration (Fig 1). A shave biopsy revealed a hyperplastic epidermis populated by large, pale, “pagetoid” cells with atypical nuclei extending into adnexal structures (Fig 2). Immunohistochemistry showed positivity for cytokeratin 7 (CK7) (Fig 3) and p63 but negativity for antihuman epithelium antigen (Ber-EP4), suggestive of a diagnosis of ectopic eEMPD of primary skin origin. Following surgical excision with peripheral and deep en face margin assessment, which showed clear deep and peripheral margins, the patient was advised to undergo regular dermatological follow-ups and was referred to an oncologist for a comprehensive systemic evaluation.

## Case report 2

**Fig 4:**
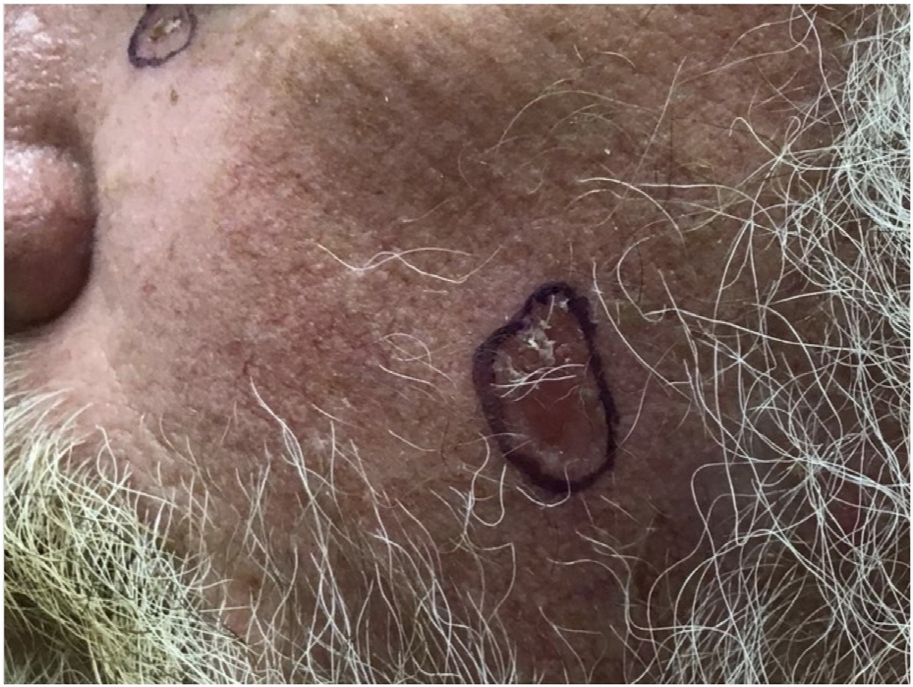
A 1.4 cm slowly enlarging, mildly pruritic, erythematous, and scaling patch on left cheek from Case 2 that was initially diagnosed at melanoma in situ (MIS).

**Fig 5:**
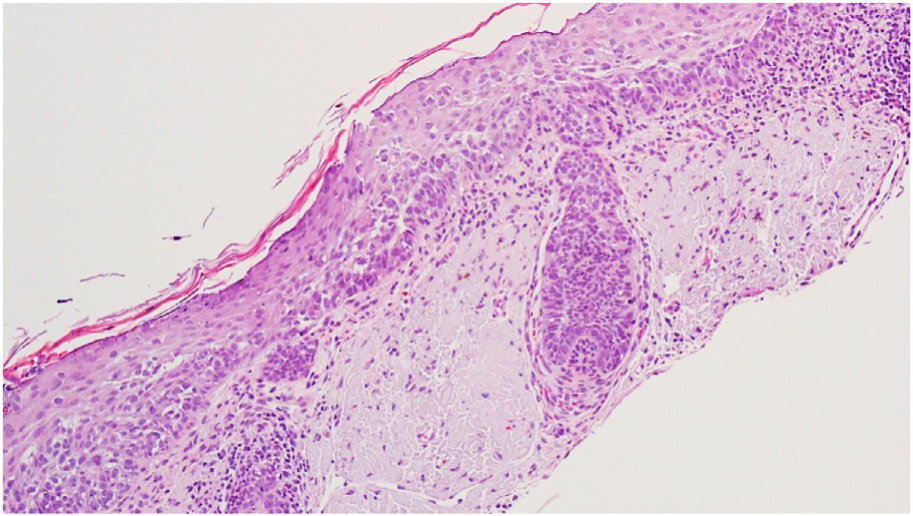
Shave biopsy from Case 2 initially diagnosed as melanoma in situ (MIS) showing an atypical lentiginous proliferation of cells arranged singly and in nests with prominent pagetoid scatter (H&E 20×).

**Fig 6:**
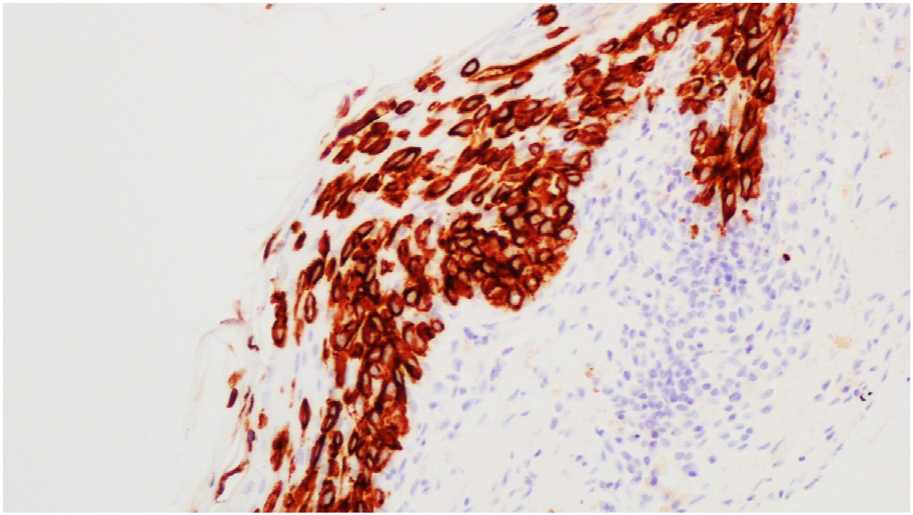
Immunohistochemical staining from Case 2 showing strong positivity for cytokeratin 7 (CK7).

A 62-year-old male with Fitzpatrick skin type II presented with a slowly enlarging, mildly pruritic, erythematous, scaling, 1.4 cm patch on his left cheek (Fig 4). Initially diagnosed as melanoma in situ via shave biopsy (Fig 5), further excisional analysis with peripheral and deep en face margin assessment revealed atypical epithelioid cells with clear cytoplasm, organized in nests and scattered throughout the epidermis. Immunohistochemical staining showed positivity for CK7 (Fig 6) and negativity for Melan A, p63, Preferentially Expressed Antigen in Melanoma, and Ber-EP4, leading to a revised eEMPD diagnosis. The patient underwent systemic evaluation, including a negative mammogram and a computed tomography scan that identified an incidental lung nodule. The patient was monitored with a follow-up scan, which was recommended in 1 year. No adjuvant therapy was advised, but referral to a major academic research center for clinical trial consideration was made, alongside regular dermatological follow-ups to monitor for recurrence.

## Discussion

Extramammary Paget’s disease can represent an intraepidermal adenocarcinoma possibly arising from Toker cells, intraepidermal extension of an adnexal carcinoma from either apocrine glands or underlying mammary-like glands, or an epidermotropic metastasis from underlying adenocarcinoma.^10^ In Caucasian populations, EMPD is most commonly found in women with an average age of 65 at diagnosis.^2^^,^^7^ Conversely, in Asian populations, EMPD has a male preponderance with a ratio of 3.5:1.^4^^,^^8^ The term eEMPD refers to exceptionally rare cases of EMPD lesions found in areas distant from the common apocrine gland-rich anatomic areas, such as the axillary, perianal, and genital regions.^1^^,^^2^^,^^10^

Clinically, eEMPD usually presents as a pruritic, velvety, soft, red/pink patch or plaque with overlying white hyperkeratosis and surrounding erosions—often referred to in the medical literature as “strawberries and cream.”^1^, ^2^, ^3^ The clinical differential diagnosis encompasses squamous cell carcinoma, fungal infections, pagetoid basal cell carcinoma, eczema, psoriasis, contact dermatitis, lichen sclerosus, mycosis fungoides, and melanoma.^2^^,^^5^

Histologically, EMPD is characterized by epidermal Paget cells with abundant clear cytoplasm and a pleomorphic hyperchromatic nucleus, whose appearance often prompts a “pagetoid scatter” differential, including: squamous cell carcinoma in situ, Merkel cell carcinoma, sebaceous carcinoma, Paget’s Disease, and melanoma amongst others.^8^ Therefore, it can be challenging to discern definitively between these diagnoses without using immunohistochemical stains to differentiate cellular lineage, such as p63, S100, HMB45, cytokeratin antibody marker 5.2, CK7, acidic cytokeratin, basic cytokeratin, CEA, CK20, and GCDFP.^8^ EMPD has also been found to have a high sensitivity for Ber-EP4 when used to compare pagetoid squamous cell carcinoma *in situ* and pagetoid melanoma *in situ* which is contrary to the findings seen in the 2 cases presented here. However, Ber-EP4 it is not considered specific as it can stain basal cell carcinomas and Merkel cell carcinoma.^9^ This discrepancy may be due to variability in the antibody clone chosen for use, processing methods that affected staining outcomes, or aberrant staining of squamous cells with CK7.

After excluding other possibilities, the next step involves determining whether the EMPD is a primary neoplasm or a secondary neoplasm arising from an underlying malignancy. Primary EMPD stains positive for CK7, GCDFP, and polyclonal CEA. EMPD that stains positive for CK20 and negative for GCDFP suggests the EMPD is secondary to underlying visceral malignancy, thus necessitating a systemic workup.^2^^,^^4^, ^5^, ^6^ More recently, in their investigation of 72 cases of primary EMPD published in 2017, *Zhao et al* found that GATA3, a well-known marker for breast carcinoma, is actually more sensitive than GCDFP for primary genital EMPD.^11^

Both ectopic and ordinary EMPD lack any significant differences in clinical presentation save for location.^1^^,^^4^ Wide local excision was historically the standard of care for managing ectopic and ordinary EMPD, with a suggested margin between 2 cm and 3 cm.^1^ However, Mohs micrographic surgery has since overtaken wide local excision as the standard of care due to its lower recurrence rates. If the patient is a poor surgical candidate, other nonsurgical treatment modalities are effective such as radiotherapy, photodynamic therapy, or topical imiquimod cream, but have higher recurrence rates.^1^

In their review article, *Scarbrough et al* note that eEMPD appears to have a more benign biological activity when compared to EMPD and cite only 1 associated case in the medical literature of lymph node metastasis. Nevertheless, *Scarbrough et al* recommend that the treatment plan for eEMPD include a focused screening for underlying malignancy.^1^ Due to the high recurrence rate of EMPD being in the range of 31% to 61% after surgical excision, regular follow-up with a dermatologist is recommended to monitor for recurrence clinically.^3^

## Conflicts of interest

None disclosed.
